# Dissection of keratin network formation, turnover and reorganization in living murine embryos

**DOI:** 10.1038/srep09007

**Published:** 2015-03-11

**Authors:** Nicole Schwarz, Reinhard Windoffer, Thomas M. Magin, Rudolf E. Leube

**Affiliations:** 1 Institute of Molecular and Cellular Anatomy, RWTH Aachen University, Aachen, Germany; 2 Translational Center for Regenerative Medicine and Institute of Biology, University of Leipzig, Leipzig, Germany

## Abstract

Epithelial functions are fundamentally determined by cytoskeletal keratin network organization. However, our understanding of keratin network plasticity is only based on analyses of cultured cells overexpressing fluorescently tagged keratins. In order to learn how keratin network organization is affected by various signals in functional epithelial tissues *in vivo*, we generated a knock-in mouse that produces fluorescence-tagged keratin 8. Homozygous keratin 8-YFP knock-in mice develop normally and show the expected expression of the fluorescent keratin network both in fixed and in vital tissues. In developing embryos, we observe for the first time *de novo* keratin network biogenesis in close proximity to desmosomal adhesion sites, keratin turnover in interphase cells and keratin rearrangements in dividing cells at subcellular resolution during formation of the first epithelial tissue. This mouse model will help to further dissect keratin network dynamics in its native tissue context during physiological and also pathological events.

Keratins are among the most abundant proteins in epithelial tissues providing resistance to environmental stress. Their essential contribution to epithelial function is attested to by a broad range of keratin-related human diseases with barrier defects, inflammation, hyperproliferation and dedifferentiation and by a growing number of murine knockouts with pronounced epithelial dysfunction^1^,^2^,^3^. Keratin function is considered to be dependent on a high degree of compositional and structural plasticity of the keratin cytoskeleton. The more than 50 keratin polypeptides assemble as obligatory type I/type II heterodimers, which are expressed in a context-specific manner^4^,^5^. While assembly of keratin heterodimers into 10 nm filaments occurs spontaneously without proteinaceous co-factors or nucleoside triphosphates *in vitro*, *in vivo* assembly of keratins into desmosome-anchored networks appears to be much more complex. So far, our knowledge of keratin network plasticity has been restricted to analyses of cultured cells that overexpress fluorescence-tagged keratins^6^,^7^. It has been proposed that the observed dynamic processes are part of a spatially defined multistep turnover cycle^2^,^7^. How keratin networks become organized and react to various signals in functional epithelial tissues is, however, completely unknown.

To address these questions, we wanted to generate a knock-in mouse producing fluorescence-tagged keratins, which mimic the properties of endogenous keratins as closely as possible. We selected enhanced yellow fluorescent protein (YFP) as a tag because of its superior brightness and stability^8^ in combination with low *in vivo* toxicity^9^. As a suitable target we chose the keratin 8 gene (*Krt8*) because it is expressed in all simple epithelia, in certain compartments of all multilayered epithelia, and in most carcinomas^4^,^5^. Furthermore, along with keratin 7, it is the first intermediate filament protein to be synthesized during embryogenesis^10^,^11^,^12^,^13^,^14^,^15^.

## Results

### Establishment of healthy homozygous Krt8-YFP knock-in mice

**Figure 1:**
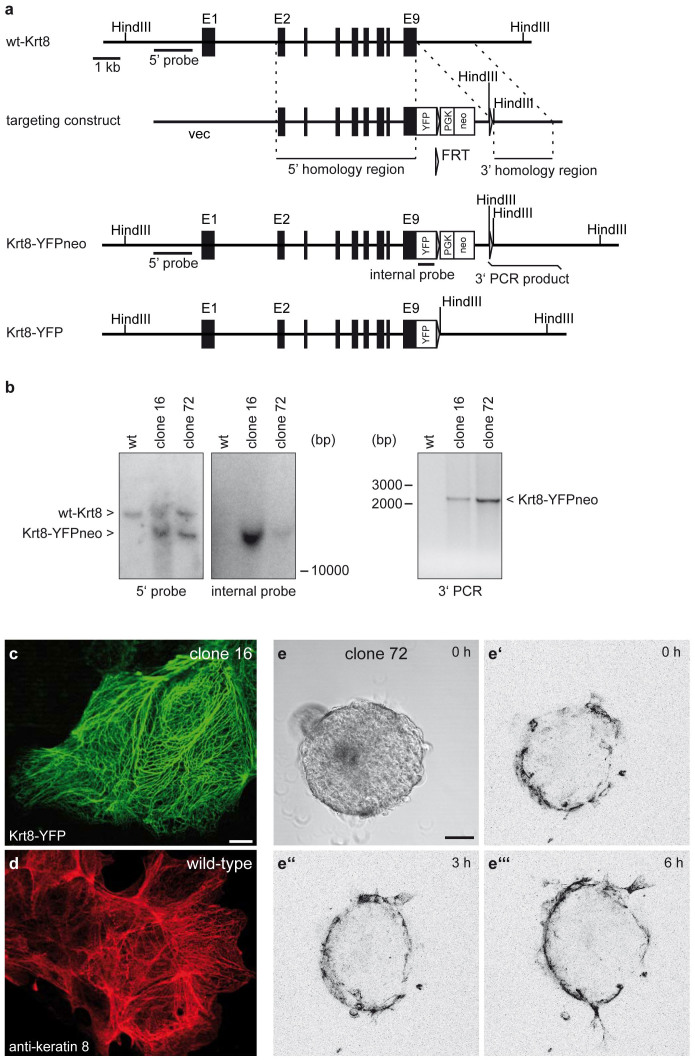
Generation of Krt8-YFP knock-in embryonic stem cells. (a) Scheme illustrating strategy to insert YFP-encoding cDNA in frame into exon 9 of the wild-type *Krt8* gene (wt-Krt8). The intron/exon structure of *Krt8* is depicted with exons E1-E9 (black boxes). The targeting construct shown below consists of a vector backbone (vec), a 4.1 kb 5′ homology region, an YFP-encoding cassette (YFP), a phosphoglycerate kinase promoter (PGK)-driven neomycin resistance cassette (neo), flanked on both sites by Flp recombinase recognition sites (FRT), and a 2.1 kb 3′ homology region. Recombination of the targeting construct with the wild-type *Krt8* allele is expected to result in knock-in allele Krt8-YFPneo. Subsequent Flp-mediated excision of the PGK-neo module leads to allele Krt8-YFP. (b) Autoradiographs of Southern blot hybridization experiments (left) and picture of ethidium bromide-stained PCR products (right) detecting DNA fragments of wt-Krt8 and Krt8-YFPneo. Genomic DNA was prepared from wild-type embryonic stem cells (ESCs, wt) and ESC clones 16 and 72, which were obtained after transfection with the targeting construct and G418 selection. DNA was digested with HindIII prior to gel electrophoresis for Southern blot hybridization with either a 5′ or an internal probe (a). To test for correct 3′ integration, PCR was performed with primers located at the end of the PGK-neo module and 50 bp downstream of the 3′ homology region generating a 2.2 kb product from the recombinant Krt8-YFP allele. Complete blots in Supplementary Figure 2. (c–d) Fluorescence microscopy of Krt8-YFPneo-containing differentiating clone 16 ESCs (c) and indirect immunofluorescence microscopy using anti-keratin 8 antibodies in differentiating wild-type ESCs for comparison (d). Note that the extended cytoplasmic keratin networks are very similar. Scale bar, 10 μm in c (same magnification in d). (e–e′′′) Fluorescence micrographs taken from a time-lapse recording (Supplementary Movie 1) and corresponding phase contrast image at time point 0 of an embryoid body derived from ESC clone 72. The Krt8-YFP fluorescence is restricted to the outer cell layer corresponding to the primitive endoderm. Note that some of the highly motile cells migrate outward to the gelatin-coated glass-bottom dish upon attachment of the embryoid body. Scale bar, 50 μm.

The YFP-encoding gene was inserted in frame at the end of the protein-coding sequence in exon 9 of the *Krt8* gene by homologous recombination in embryonic stem cells (ESCs) as detailed in Fig. 1a. The insertion should not adversely affect transcription of the Krt8-coding sequence since all of the native gene regions are maintained in the recombined gene locus. Correct integration of the targeting construct into the *Krt8* gene was verified by PCR and Southern blotting in selected ESC clones (Fig. 1b). Fluorescence microscopy was then performed to examine the expression of keratin 8-YFP (Krt8-YFP) in fixed differentiating ESCs. The comparison of the images in Fig. 1c and Fig. 1d shows that the fluorescent Krt8-YFP filament network in recombinant ESCs is similar to that observed by anti-keratin 8 staining in differentiating wild-type ESCs. Fluorescence microscopy of vital embryoid bodies derived from Krt8-YFP knock-in ESCs revealed a restricted expression of Krt8-YFP in the outer cell layer (Fig. 1e,e′), which is known to be keratin 8-positive^16^ and corresponds to the primitive endoderm^17^. Time-lapse fluorescence microscopy could be performed for extended periods on embryonic bodies. The example presented in Fig. 1e′–e′′′ and corresponding Supplementary Movie 1 depicts the substantial rearrangements of the keratin network during the cell cycle and in the highly motile outgrowing cells.

Correctly targeted ESCs were subsequently used to generate Krt8-YFP chimeric mice. The recombinant Krt8-YFPneo allele (Fig. 1a) was transmitted through the germline to transgenic offspring. These animals were subsequently crossed with the FLP° deleter strain^18^. The neomycin resistance cassette was successfully removed by flp-mediated recombination as determined by PCR in the resulting offspring. Homozygous and heterozygous Krt8-YFP knock-in mice were born to heterozygous parents close to the expected Mendelian ratio (wildtype: 31%; heterozygous knock-in: 45%; homozygous knock-in: 24%; n = 42). All knock-in mice developed normally, demonstrating that the Krt8-YFP fusion protein functionally substitutes for wild-type keratin 8, which, depending on the genetic background, has been shown to be essential for embryogenesis and intestinal function^19^,^20^. Matings of homozygous mice resulted in 5.6 pups/litter (n = 139), which is in the expected range for the genetic background of the mouse strain (C57BL/6 and 129/Ola; http://www.informatics.jax.org/). The sex distribution of the offspring was also within the normal range (45% males). No obvious defects of keratin 8 expressing tissues were detected in hematoxylin-eosin-stained sections of homozygous Krt8-YFP knock-in mice (Supplementary Figure 1).

### Orthotopic localization of Krt8-YFP in fixed and vital tissues of adult mice

**Figure 2:**
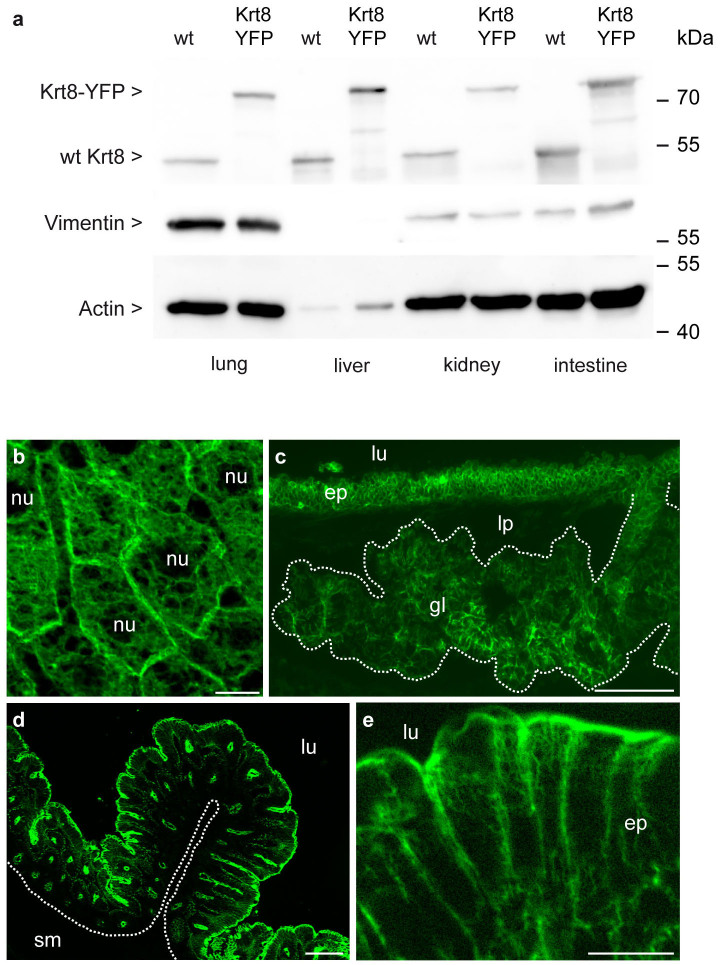
Detection of Krt8-YFP by immunoblotting and fluorescence microscopy in tissues of adult knock-in mice. (a) Immunoblots of cytoskeletal extracts that were prepared from lung, liver, kidney and intestine of wild-type (wt) and homozygous Krt8-YFP mice (Krt8-YFP). Blots were successively incubated with anti-keratin 8 antibodies (top), anti-vimentin antibodies (middle) and anti-actin antibodies (bottom). Note the increase in the size of keratin 8 due to the YFP tag and the complete absence of wild-type keratin 8 in the homozygous Krt8-YFP knock-in mouse samples. Full-length blots are presented in Supplementary Figure 2. (b–e) Fluorescence microscopy of cryosections of liver (b), trachea (c) and intestine (d, e) obtained from homozygous Krt8-YFP mice. Note the exclusive expression of the transgene in the cytoplasm of epithelial cells but not in the nucleus (nu) nor in connective tissue of the lamina propria (lp; c) and submucosa (sm; d). Note also the cell type-specific network organization with a pancytoplasmic distribution in adluminal cells of the tracheal epithelium (c; ep, epithelium) and in glandular epithelial cells (c; gl, gland), the accumulation around bile canaliculi in the liver (b) and subapical enrichment in intestinal epithelial cells (d, e; ep, epithelium). lu, lumen. Scale bars, 10 μm in (b, e) and 100 μm in (c, d).

According to previous reports on keratin 8 expression^4^,^5^ Krt8-YFP is expected to be produced in simple and complex epithelia. Cytoskeletal extracts were therefore prepared from lung, liver, kidney, and intestine of adult knock-in mice to detect Krt8-YFP by immunoblotting. Fig. 2a shows that Krt8-YFP fusion proteins with the expected molecular mass of approximately 82 kDa are present in these tissues of homozygous knock-in mice at levels comparable to those observed for wild-type keratin 8 in control animals. We next examined Krt8-YFP fluorescence in cryosections of liver, trachea and intestine (Fig. 2b–e). Krt8-YFP was restricted to the cell types known to express keratin 8^4^. In addition, network organization such as accumulation around bile canaliculi in hepatocytes and subapical location in enterocytes was identical to that reported for the wild-type situation^21^,^22^,^23^.

**Figure 3:**
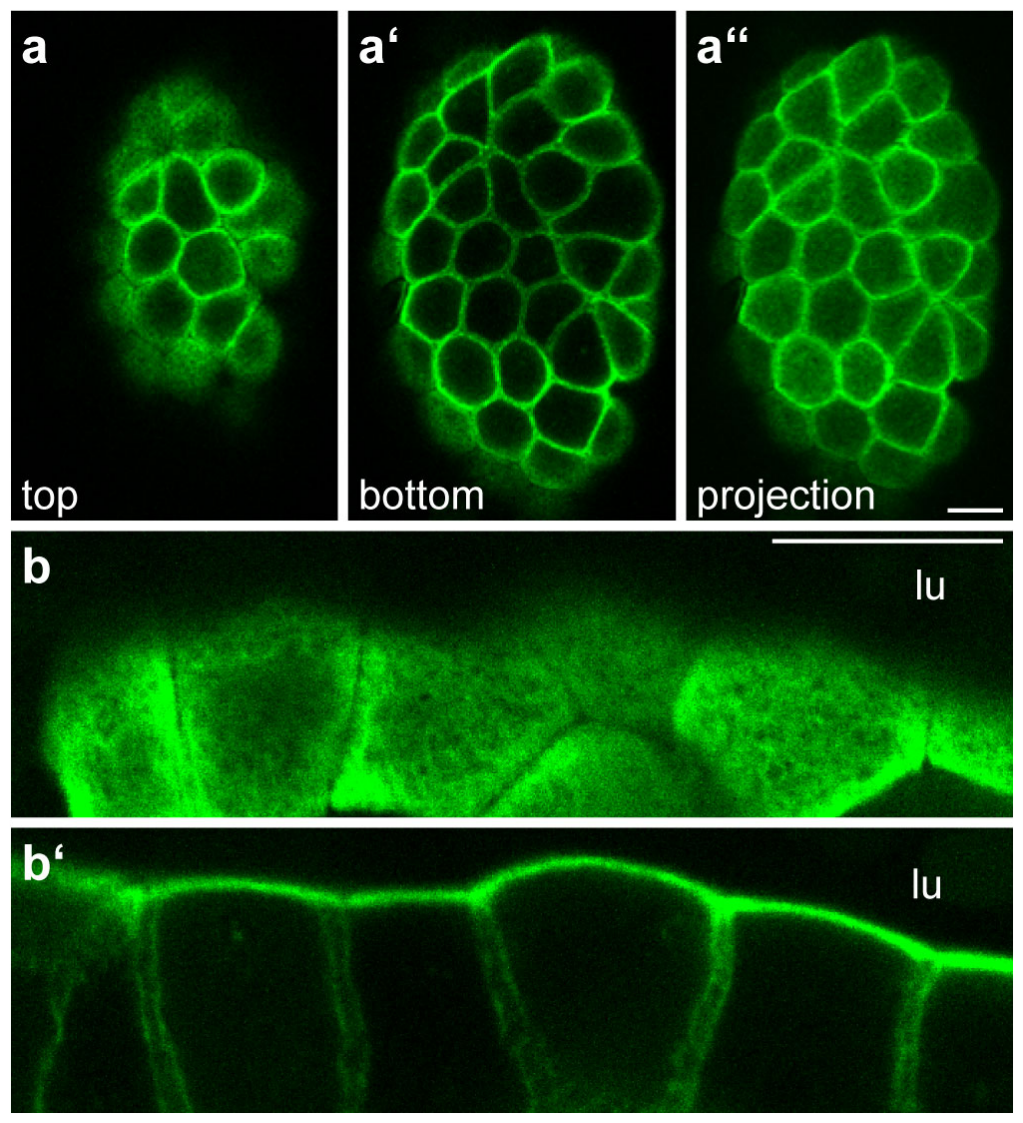
Detection of Krt8-YFP by fluorescence microscopy in living intestinal mucosa. (a–a′′) Two different z planes (a, a′) from a stack of 19 focal planes (maximum intensity projection of all planes in a′′) are shown to depict the subapical dense network in the enterocytes and the laterally restricted filaments below (see also corresponding animation in Supplementary Movie 2). (b–b′) Images taken from a high-resolution stack of 13 focal planes showing an oblique surface views of the dense subapical network (b) and the network below that is restricted to the cortical juxtamembraneous domain of adjacent intestinal epithelial cells (b′; complete image series in Supplementary Movie 3). lu, lumen. Scale bars, 10 μm.

To test the suitability of the Krt8-YFP reporter for *in vivo* imaging, we examined freshly prepared, non-fixed intestinal mucosa. The resulting fluorescence images (Fig. 3a–b′; Supplementary Movies 2, 3) revealed details that were clearly superior to those obtainable by standard immunofluorescence techniques. Examples include the dense subapical network and the clearly distinguishable filament bundles below the lateral membranes of adjacent cells.

### De novo keratin network formation in close proximity to desmosomes of pre-implantation embryos

**Figure 4:**
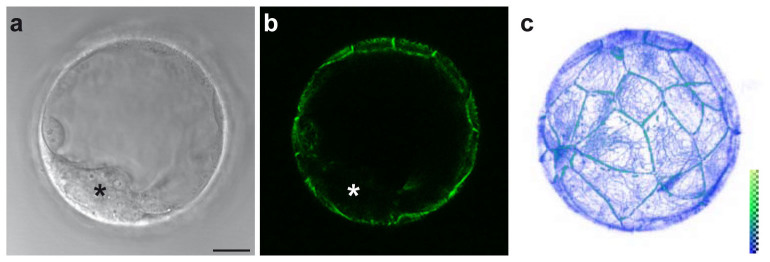
Trophectoderm-restricted expression of Krt8-YFP in a homozygous Krt8-YFP late blastocyst. (a, b) Brightfield image and corresponding fluorescence image showing Krt8-YFP exclusively in the outer trophectoderm layer, while the inner cell mass is completely negative (asterisk). (c) 3D-reconstruction of the fluorescence recording of 72 focal planes (1 μm steps) of the blastocyst shown in a. The relative fluorescence intensity is color coded. An animation of the reconstruction is presented in Supplementary Movie 4. Scale bar, 20 μm.

To prove that the Krt8-YFP knock-in mice can be used to monitor keratin network formation and dynamics at high resolution *in vivo*, we decided to examine pre-implantation embryos. They are ideally suited for imaging due to their transparency, small size and the external location of their epithelial trophectodermal cells, which undergo rapid division during development. Imaging of live blastocysts revealed the expected trophectoderm-restricted keratin filament network (compare^11^ with Fig. 4). The 3D-animation in Supplementary Movie 4 further highlights the high degree of detail of the nascent cytoplasmic keratin network and prominent accumulations at cell-cell borders that can be achieved.

**Figure 5:**
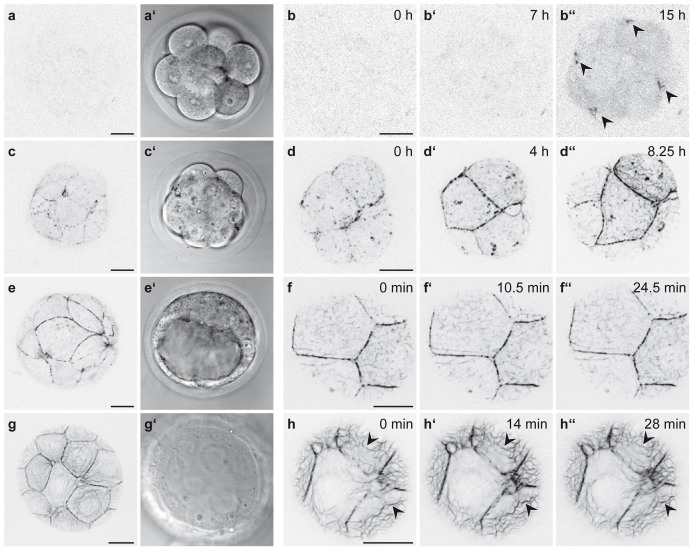
Keratin network morphogenesis in Krt8-YFP embryos. (a, a′) Fluorescence and brightfield of 8-cell stage shows complete absence of Krt8-YFP. (b–b′′) Increasing diffuse fluorescence and appearance of fluorescent dots (b′′; arrowheads) is observed 7-8 hours after compaction (b′). Images are taken from Supplementary Movie 5. (c–c′) Fluorescence and brightfield of early Krt8-YFP blastocyst reveal dotted keratin fluorescence at cell borders and cytoplasmic keratin particles. (d–d′′) The fluorescence micrographs are taken from Supplementary Movie 6 and depict keratin dynamics from the early to the mid-blastula stage. Note the increasing fluorescent dots at the cell-cell borders and the appearance of cytoplasmic keratin particles and filaments. (e–e′) Fluorescence and brightfield microscopy of mid-blastocyst shows that the Krt8-YFP positive puncta become interconnected by filamentous structures. (f–f′′) Micrographs from Supplementary Movie 7 demonstrate the overall changes in distribution and arrangement of keratin particles in contrast to the stable arrangement of elongated keratin filaments and juxtamembraneous puncta. (g–g′) Krt8-YFP fluorescence and brightfield microscopy of a late blastocyst reveals an extensive cytoplasmic keratin network anchored to cell borders. (h–h′′) Images from Supplementary Movie 8 depict overall keratin network stability (arrowheads). All scale bars, 20 μm.

**Figure 6:**
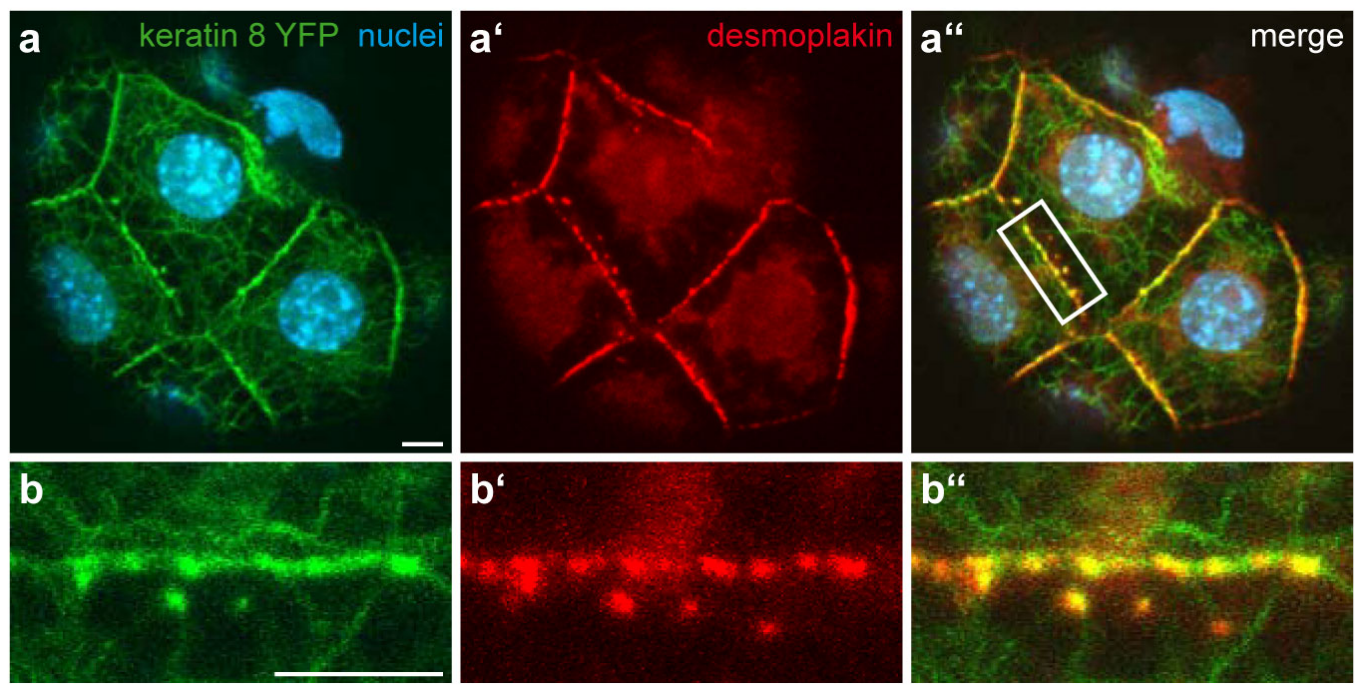
Co-localisation of desmoplakin and Krt8-YFP in a blastocyst. (a–a′′) Fluorescence microscopy of Krt8-YFP distribution with Hoechst33342-staining to detect nuclear DNA (a) and anti-desmoplakin staining (a′) in the trophectoderm of a fixed homozygous Krt8-YFP blastocyst. The merged image shows co-localization of Krt8-YFP and desmoplakin (a′′). (b–b′′) Details of membrane staining (white box in a′′). Note the co-distribution of fluorescent keratins and desmoplakin-positive structures at cell-cell borders. All scale bars, 5 μm.

To document *de novo* keratin network formation, we imaged free-floating homozygous Krt8-YFP embryos. As expected, 8-cell stage embryos did not show any fluorescence signal until after they had compacted^12^ (Fig. 5a–a′, b, b′). Six to 8 hours after compaction, a diffuse cytoplasmic Krt8-YFP signal appeared and strongly fluorescent dots formed that accumulated at cell borders (Supplementary Movie 5; arrowheads in Fig. 5b′′). During the subsequent maturation of the compacted morula (Supplementary Movie 6; Fig. 5c–e′), the dotted signal at cell borders increased continuously. At the same time, fluorescent dots emerged throughout the cytoplasm and subsequently elongated into filamentous particles, giving rise to unstable reticular structures, predominantly in the cell periphery. Images recorded of mid-blastocysts at higher spatial and temporal resolution (Supplementary Movie 7; Fig. 5f–f′′) revealed that the cytoplasmic particles were highly flexible and moved around randomly. Occasionally, particles fused with each other into longer filaments that sometimes broke apart again. Gradually, a fully developed keratin filament network appeared (Fig. 5g–g′). The filaments within the network were stably integrated with only minor fluctuations (Supplementary Movie 8; arrowheads in Fig. 5h–h′′). The dotted cell border fluorescence persisted but became less prominent in comparison to the increasing cytoplasmic filament network. In addition, filamentous elements appeared that interconnected the dots. Immunofluorescence microscopy further revealed that the dots co-localized perfectly with the desmosomal marker desmoplakin (Fig. 6).

### Turnover of juxtamembraneous keratins in living blastocysts and keratin dynamics during cell division in developing murine embryos

**Figure 7:**
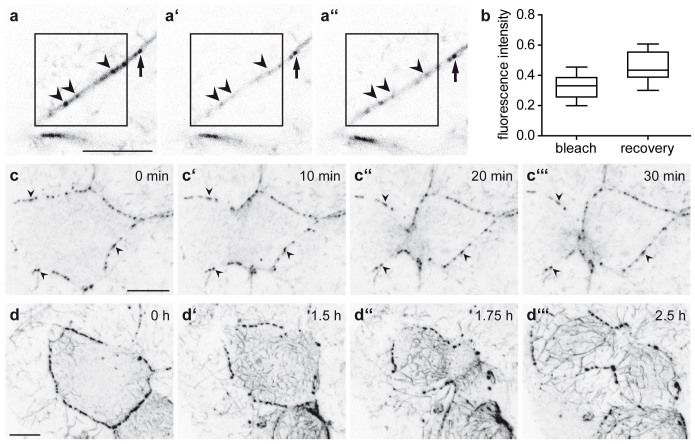
Keratin network turnover and rearrangement in Krt8-YFP embryos. (a–b) Fluorescence micrographs before (a) and after bleaching (0 minutes in a′ and 15 minutes in a″) of prominent keratin accumulations in dotted structures (arrowheads on reappearing dot; arrow on outside control dot) and quantification of fluorescence recovery after photobleaching experiments. Data are represented as Tukey's Whisker plots. n = 9, p < 0.0001 (two-tailed paired *t*-test). (c–d′′′) Pictures from Supplementary Movies 9 and 10 recording Krt8-YFP fluorescence in dividing trophoblast cells of blastocysts with different keratin distribution. Note persistence of dotted cell border (arrowheads) and slightly reduced cytoplasmic particle fluorescence in combination with transient increase of diffuse cytoplasmic fluorescence. Note also the rapid appearance of an extended keratin network in the late blastocyst. All scale bars, 10 μm.

To examine the turnover of keratins in their natural environment, we performed fluorescence recovery after photobleaching (FRAP) experiments. Results are shown for the strong dotted Krt8-YFP signal (Fig. 7a–b). The fluorescence signal was reduced to 32.55 ± 8.09% immediately after bleaching and gradually recovered to 46.34 ± 10% within 15 minutes. It is notable that recovery occurred preferentially at the original fluorescent sites, indicating intrinsic turnover of keratin-positive structures.

Finally, we recorded keratin rearrangements during trophectodermal cell divisions in early and late blastocysts (Fig. 7c–c′′′, Supplementary Movie 9 and Fig. 7d–d′′′, Supplementary Movie 10, respectively). The dotted fluorescence persisted throughout mitosis in each instance. Diffuse cytoplasmic fluorescence increased upon entry into mitosis and was cleared at the end of mitosis. While the motile particles remained in the cytoplasm of the daughter cells in early blastocysts, daughter cells at the late blastocyst stage established extensive keratin filament networks soon after cleavage. In this instance, we also noted reversible granule formation, as has been described for cultured cells^24^.

## Discussion

We introduce a novel knock-in reporter mouse expressing keratin 8-YFP instead of the endogenous keratin 8 as a versatile tool to study intermediate filament network biogenesis and dynamics *in vivo*. Homozygous keratin 8-YFP knock-in mice are viable and are born at the expected Mendelian ratio. The tagged keratin is obviously functional since keratin 8 knock-out mice die during embryogenesis or develop intestinal lesions depending on the genetic background^19^,^20^. We furthermore demonstrate that the cell type-specific expression and distinct subcellular localization patterns of the Krt8-YFP fusion protein are indistinguishable from those observed in the wild-type situation^4^,^14^,^21^,^22^,^23^. Since fixation of samples can be avoided altogether, superior resolution can be obtained in cells and tissues of the Krt8-YFP mice. Most importantly, changes in network organization can be tracked over time in a functional tissue context. Thus, imaging of developing pre-implantation embryos provided novel insights into the dynamics of cytoplasmic intermediate filaments in their native environment at physiological expression levels. Some of our observations are in accordance with previously reported *in vitro* observations while others are not. For example, the turnover rates of keratin filaments^7^,^25^,^26^ as well as the fusion competence and high flexibility of elongated cytoplasmic keratin particles are similar^25^,^27^,^28^. On the other hand, the random movement of keratin particles *in vivo* appears to be different from the ordered microtubule- and actin-dependent motility observed in cultured cells^6^,^28^,^29^. Furthermore, the close correlation between desmosomes and keratin network formation has not been observed by time-lapse fluorescence microscopy in cultured cells, although it has been known for a long time that desmosome biogenesis and keratin network morphogenesis occur during the same developmental period^12^,^30^. Our new data revive the old idea that desmosomes act as organizing centers for keratin filament networks^31^,^32^.

The novel mouse model opens up an entirely new avenue for examining cell type-specific keratin filament organization and for studying *in vivo* responses of the keratin cytoskeleton to microbial, chemical and physical insults in epithelial cells. The observed transitions from keratin-negative ESCs to keratin-positive outer cells in embryoid bodies, from keratin-negative 8-cell stage blastomeres to keratin-positive morula blastomeres and from keratin-positive morula blastomeres to keratin-negative inner cell mass cells, all demonstrate the value of the novel mouse line to track reversible transitions between epithelial and mesenchymal phenotypes *in vivo*, which is important for the understanding of carcinogenesis.

In summary, we have generated a new fluorescent reporter mouse, which, for the first time, allows formation, turnover and mitotic rearrangement of keratin networks to be faithfully monitored in a native tissue context throughout the cell cycle. This is therefore the first report on intravital recording of a cytoskeletal component in developing pre-implantation embryos. Improvements in instrumentation (e.g., light sheet microscopy) will help to further increase the already high level of resolution. Thus, intravital examination of fluorescent reporters during early embryogenesis will help to unravel many fundamental problems in tissue morphogenesis under physiological conditions with manifold options for controlled manipulation.

## Methods

### Preparation of targeting construct

To prepare the Krt8-YFP targeting vector, the YFP-cDNA was PCR-amplified from plasmid pEYFP-N1 (Clontech) with amplimers #07-052 5′-ATC GCG GCC GAT GGT GAG CAA GGG CGA GG-3′ and #07-053 5′-ATC GAC TAG TTT ACT TGT ACA GCT CGT CCA T-3′ and was first subcloned into pJet1.2/blunt (CloneJET PCR Cloning Kit, ThermoScientific) and then into pBluescript(KS+) using EagI and SpeI restriction sites giving rise to plasmid #3071.

The 2.1 kb 3′ homology region was amplified from genomic DNA, which was isolated from HM-1 embryonic stem cells^33^, using amplimers #07-054 5′- ATC GAA GCT TAA CTA GCT TTG CTA TGT ATG C-3′ and #07-055 5′- ATC GCT CGA GCA AAC TGA AAA TAT TGA CTT AGG-3′. The resulting fragment was digested with HindIII and XhoI and inserted into plasmid #3071 giving rise to plasmid #3073.

To clone the 5′ homology fragment, a linker containing an additional NcoI site was first inserted into the EagI site of pBluescript(KS-) using oligonucleotides #07-089 5′- GGC CAT CCA ACC ATG GAA CCT C-3′ and #07-090 5′-GGC CGA GGT TCC ATG GTT GGA T-3′ giving rise to plasmid #3075. The 5′ homology region was then amplified and cloned in several steps. First, a 2.6 kb fragment was amplified from HM-1 cell-derived genomic DNA by PCR using primers #07-078 5′- TGT CAA CCA TGG GCA GAT G-3′ and #07-057 5′-ATC GCG GCC GAC ACT TGG ACA CGA CAT CAG-3′ and subcloned into pJet1.2/blunt giving rise to plasmid #3074. This plasmid was cut with NcoI and EagI and the 2.6 kb fragment was subcloned into the corresponding sites of plasmid #3075 to obtain plasmid #3077. Another PCR was performed on genomic DNA from HM-1 cells using primers #07-092 5′-ATC GCC GCG GCT CCA GAT CCT GAC TAT AGC C-3′ and #07-079 5′- CTG CCC ATG GTT GAC ACT TG-3′ producing a 2.1 kb fragment that was cloned into pJet2.1/blunt. It was subsequently subcloned into #3075 using SacII and NcoI restriction sites giving rise to plasmid #3078. Plasmid #3077 was cut with EagI and NcoI and the 2.6 kb fragment was inserted into #3078 producing plasmid #3079 encompassing the fused 4.7 kb 5′ homology fragment. Plasmid #3079 was cut with EagI and SacII and the resulting 4.7 kb fragment was inserted into plasmid #3073 that contained the YFP sequence and the 3′ homology region giving rise to plasmid #3084.

To amplify the "NFL box" consisting of two Frt-sites flanking the phosphoglycerate kinase promoter sequence next to a neomycin resistance-encoding cassette, a PCR using primers #07-058 5′-ATC GCC CGG GGC TGC GAT TAT AGG CCT GAG-3′ and #07-059 5′-ATC GCC CGG GGG GAT AAT ACG ACT CAC TAT AG-3′ was performed on plasmid #3011^34^. The resulting 2 kb product was inserted into #3084 using the XmaI restriction site giving rise to the final Krt8-YFP targeting vector (#3085). It contains the 4.1 kb 5′ homology region spanning exon 2 to exon 9 of the *Krt8* gene followed by the YFP sequence, the NFL box and the 2.1 kb 3′ region of the murine *Krt8* locus.

### Cell culture and selection of recombinant cell clones

HM-1 murine embryonic stem cells^33^ were kept in Glasgow Minimum Essential Medium (GMEM, Invitrogen) supplemented with 1 mM sodium pyruvate (PAA), 1 000 U/ml leukemia inhibitory factor (LIF, Sigma), 2 mM L-glutamine (PAA), 1× non-essential amino acids (PAA), 10^−4^ M beta-mercaptoethanol (Sigma), 15% fetal calf serum (PAA) on irradiated 3T3 (ATCC) feeder cells in gelatinized culture flasks (BD). For homologous recombination 25 × 10^6^ cells were electroporated (3 μF, 800 V) with 200 μg XhoI-linearized DNA of plasmid #3085 in Hank's Balanced Salt Solution (HBSS, Gibco) and plated at 2 × 10^6^ cells/10 cm culture dish afterwards. Selection for recombinant clones was started the next day with 350 μg/ml G418 (PAA). Resulting colonies were passaged and first analyzed by PCR for correct 3′ insertion with primers #08-017 5′-AAT TCG CCC TAT AGT GAG TCG-3′ and #08-018 5′-TGG CAA CGG GAC CTG ATA AG-3′. Clones that were tested positive were subsequently examined by Southern blot hybridization. 30 μg genomic DNA were digested with HindIII, separated on a 0.7% agarose gel, depurinated in the gel by incubation in 0.25 M HCl for 20 minutes and subsequently denatured by treatment with 0.4 M NaOH. DNA was then transferred onto a neutral nylon membrane (Hybond-N, GE Healthcare) overnight in 0.4 M NaOH and the membrane was dried for 1 hour at 80°C. A 5′ probe (1020 bp) was amplified from genomic DNA by PCR with primers #09-096 5′-AGA GAC AGG TGC AGG GAG AC-3′ and #09-097 5′-CCT GGG ATC TCT GAC CTA AG-3′. An internal probe (602 bp) was prepared from #3085 using primers #08-029 5′-TGG ACG GCG ACG TAA AC-3′ and #08-030 5′-CAT GTG ATC GCG CTT CTC-3′. The amplified fragments were labeled with α^32^P-dCTP (Perkin Elmer) using the Ladderman DNA Labeling Kit (Takara). Labeled probes were purified with the help of Illustra ProbeQuant G-50 MicroColumns (GE Healthcare). Hybridization was carried out at 65°C overnight in buffer containing 0.5 M Na_2_HPO_4_ (pH 7.2), 7% sodium dodecylsulfate (SDS), 1 mM ethylene diamine tetraacetic acid (EDTA), 100 μg/ml denatured salmon sperm DNA and 200 ng of denatured and labeled probe. Membranes were then rinsed with washing buffer 1 (2× SSC [saline-sodium citrate buffer; 20× SSC: 3 M sodium chloride, 300 mM trisodium citrate, pH 7], 0.1% SDS), washed two times for 20 minutes at 65°C with washing buffer 2 (1× SSC, 0.1% SDS) and once for 30 minutes at 65°C with washing buffer 3 (0.1× SSC, 0.1% SDS). Hybridized probe was detected by X-ray film (BioMax MR Film, Kodak) exposure of the membrane.

### Transgenic mice

Recombinant embryonic stem cell clones were harvested, re-suspended in M2 medium (Sigma) and microinjected into C57BL/6 morulae. Embryos were cultured in M16 medium (Sigma) at 37°C until transfer to recipient pseudopregnant C57BL/6 foster mothers. The resulting chimeras were bred with C57BL/6 mice giving rise to brown-coated transgenic offspring. These animals were then bred with FLP° deleter mice (kindly provided by Dr. Anastassiadis, Dresden University)^18^ to remove the neomycin resistance cassette.

Mice were housed in the animal facility of the RWTH Aachen University Hospital. All experiments were performed in accordance with the approved guidelines for the care and use of laboratory animals and licensed by Landesamt für Natur, Umwelt und Verbraucherschutz (LANUV), Nordrhein-Westfalen, Recklinghausen, Germany (reference number 11216A4).

For genotyping DNA was prepared by incubating tail tip biopsies overnight in extraction buffer (50 mM tris(hydroxymethyl)aminomethane (Tris) pH 8, 100 mM EDTA, 100 mM NaCl, 1% SDS, 0.5 mg/ml proteinase K) at 55°C. DNA was then precipitated with isopropanol from the supernatant, washed with ethanol and resuspended in TE-buffer (10 mM Tris pH 8, 1 mM EDTA). Successful removal of the neomycin resistance cassette was determined by PCR using primers #13-051 5′-GCA GCA GCA ACA AGC AGG AG-3′ and #13-052 5′- TGG ACA CCA GGG CGA AGT AG-3′ resulting in a 250 bp fragment. For genotyping PCR primers #07-088 5′-AGG AGA GCA GGT GGG TTT GG-3′ and #12-080 5′-CCC TCC CAC CTA CAC GAA TG-3′ were used to amplify a 1100 bp fragment from the wild-type Krt8 allele and a 1871 bp fragment from the Krt8-YFP (neomycin resistance cassette negative) knock-in allele.

### Immunoblot analysis

Tissue was homogenized in ice-cold low-salt buffer (10 mM Tris pH 7.5, 140 mM NaCl, 5 mM EDTA) containing 2 mM phenylmethylsulfonyl fluoride (PMSF). After centrifugation (5000 × g for 5 minutes at 4°C) the pellet was re-suspended and homogenized in ice-cold high-salt buffer (10 mM Tris pH 7.5, 140 mM NaCl, 5 mM EDTA, 1% TritonX100, 1 mM dithiothretiol (DTT), 1.5 M KCl) supplemented with 2 mM PMSF. After 30 minutes incubation on ice the samples were pelleted at 15000 × g for 10 minutes at 4°C. This procedure was repeated twice. The pellet was then re-suspended in ice-cold low salt buffer, centrifuged at 15000 × g for 10 minutes at 4°C and re-suspended in SDS sample buffer (60 mM Tris, 1.7% SDS, 8.3% glycerol, 0.34 M beta-mercaptoethanol, 0.002% bromophenol blue). Polypeptides were separated by standard SDS polyacrylamide gel electrophoresis. Proteins were transferred onto polyvinylidene fluoride Immobilon-P membrane (Millipore) by tank blotting. The membranes were then blocked with 1× RotiBlock (Roth) and incubated overnight at 4°C with rat monoclonal anti-keratin 8 (TROMA-1 hybridoma cell supernatant^11^), guinea pig monoclonal anti-vimentin (Progen) and rabbit polyclonal anti-actin (Sigma). Membranes were washed three times in TBS-T (50 mM Tris, 150 mM NaCl, 0.05% Tween 20, pH 7.6) and subsequently incubated with secondary antibodies (anti-rat HRP, anti-guinea pig HRP, anti-rabbit HRP; all from Dianova) for 1 hour at room temperature. Bound antibodies were detected with the help of ECL prime (GE healthcare) and a chemiluminescence system (Fusion SL, Vilber Lourmat). On the same membranes keratin 8, vimentin and actin were detected consecutively. Bound antibodies were stripped by incubation of the membranes with stripping buffer (100 mM glycine, pH 2) 3 times for 20 minutes.

### Histology

For fluorescence images, fresh tissue biopsies were snap-frozen in Tissue-Tek (VWR) immediately after removal. Serial 12 μm cryosections were prepared with a microtome and fixed with ice-cold acetone for 10 minutes. Sections were mounted using Mowiol 4–88 (Roth) and phase contrast and fluorescence micrographs were taken on an ApoTome.2 (Zeiss) equipped with a Plan-Apochromat 63×/1.40 Oil DIC M27 objective and an EC Plan-Neofluar 10×/0.30 Ph 1 objective.

For histological stainings, fresh tissue biopsies were fixed over night using 4% formaldehyde and embedded in paraffin. 5 μm sections were prepared with a microtome and stained with hematoxylin-eosin. Micrographs were taken on an Eclipse 80i microscope (Nikon).

### Immunofluorescence

Blastocysts were fixed with 1% PFA for 10 minutes at room temperature. Subsequently, embryos were transferred to 0.25% TritonX100 in Dulbecco's phosphate buffered saline (PBS) for 15 minutes, washed with PBS for 15 minutes and incubated with 2.6 mg/ml ammonium chloride in PBS for 10 minutes at room temperature. Blastocysts were then stored in PBS at 4°C until further use but no longer than 7 days. Blastocysts were subsequently incubated over night at 4°C with polyclonal guinea pig anti-desmoplakin antibodies (Progen) that had been diluted in PBS containing 2.5% bovine serum albumin (BSA) and 0.125% TritonX100. After washing with PBS containing 0.25% TritonX100 for 30 minutes at room temperature, blastocysts were incubated for 1 hour at room temperature with goat anti-guinea pig Alexa-633-coupled secondary antibodies (Invitrogen) that had been diluted in PBS containing 2.5% BSA and 0.125% TritonX100. Embryos were then washed with PBS for 30 minutes and incubated with 1 mg/ml Hoechst 33342 in PBS (Invitrogen) for 1 minute at room temperature. Imaging was done on free-floating blastocysts with a Zeiss LSM710Duo microscope using a 63× 1.4 N.A. DIC M27 oil immersion objective (Zeiss). For Alexa-633 fluorescence recording a 633 nm HeNe laser (Laser module LGK 7628-1F, Zeiss) was used and the emitted light was monitored between 590 nm and 660 nm. For detection of Hoechst 33342 fluorescence a diode laser (Laser cassette 405 cw, Zeiss) was used and the emitted light was monitored between 400 nm and 480 nm. The 488 nm line of an argon/krypton laser (Laser module LGK 7872 ML8, Zeiss) was used for recording of the endogenous Krt8-YFP autofluorescence and the emitted light was monitored between 500 nm and 590 nm.

Cultured ESCs growing on glass cover slides were fixed with methanol/acetone and incubated with TROMA-1 antibodies for 1 hour at room temperature. After washing with PBS for 15 minutes, cells were incubated with anti-rat Alexa-633-coupled secondary antibodies (Invitrogen) for 1 hour at room temperature, washed and mounted.

### Pre-implantation embryo isolation

Embryos were flushed out of oviducts at day E2.5 with M2 medium (Sigma) and rinsed through several drops of fresh M2 medium to remove debris. For recovery embryos were then kept for 1 hour at 37°C and 5% CO_2_ in M16 medium (Sigma) overlaid with mineral oil (Sigma).

### Live cell imaging

Embryos were individually transferred into drops of M2 medium overlaid with mineral oil in glass-bottom dishes (MatTek). Images were recorded with a Zeiss LSM710Duo microscope at 37°C. The 488 nm line of an argon/krypton laser was used for fluorescence recording via a 63× 1.4 N.A. DIC M27 oil immersion objective. The emitted light was monitored between 500 nm and 590 nm with a pinhole set at 1–2 AU (airy unit) and a laser intensity of 5%. Z-stacks were taken with intervals of 1 μm to cover approximately half the depth of the embryo. For transmitted light recordings the T-PMT detector of the Zeiss LSM710Duo was used. Image processing and analysis were performed using Zen Software (Zeiss) and Fiji. Confocal fluorescence micrographs are shown as maximum intensity projections. Only single planes of brightfield images recorded in parallel are presented for better image clarity. For 3D reconstruction of fluorescence signals AMIRA 5.5.0 (Visualization Sciences Group, SAS) software was used.

For live imaging of fluorescent keratins in embryoid bodies embryonic stem cells were grown without feeder cells and in the absence of LIF in Petri dishes (Greiner). After embryoid bodies formed, they were transferred into gelatin-coated glass-bottom dishes. Imaging was subsequently performed as described above.

For live imaging of fluorescent keratins in intestinal mucosa, colon was taken from homozygous Krt8-YFP knock-in mice and immediately flushed with pre-warmed Krebs-Henseleit buffer (114 mM NaCl, 5 mM KCl, 24 mM NaHCO_3_, 1 mM MgCl_2_, 2.2 mM CaCl_2_, 10 mM HEPES, 0.25% BSA, pH 7.35). The colon was then cut longitudinally and transferred onto glass-bottom dishes with the mucosa side towards the objective. Imaging and analysis was carried out as described above.

### Fluorescence recovery after photobleaching

Photobleaching experiments were performed with a LSM710 confocal microscope equipped with a DefiniteFocus device (Zeiss) at 37°C. The 488 nm line of an argon/krypton laser was used for both bleaching and image recording via a 63× 1.4 N.A. DIC M27 oil immersion objective. The emitted light was monitored between 500 nm and 590 nm. Bleaching in selected areas was carried out at 100% laser power for 2 scans with a pixel dwell time of 6.3 μs. The fluorescence intensity was measured at 5% laser power in the respective areas of interest prior to bleaching and directly after bleaching at 30-second intervals for 15 minutes. The gray values of the bleached and reference areas were measured in the recorded image data sets (Zen 2009, Zeiss) and analyzed using spreadsheet routines (Excel) and Prism 5 (GraphPad). Data are given as Tukey's Whisker plot and significance of differences was tested using a two-tailed paired *t*-test.

## Author Contributions

R.E.L. designed study. N.S., R.W. and T.M.M. carried out experiments. N.S., R.W., T.M.M. and R.E.L. analysed and interpreted data. N.S. and R.E.L. wrote manuscript.

## Supplementary Material

Supplementary Information Supplementary Information

Supplementary Information Supplementary Movie 1

Supplementary Information Supplementary Movie 2

Supplementary Information Supplementary Movie 3

Supplementary Information Supplementary Movie 4

Supplementary Information Supplementary Movie 5

Supplementary Information Supplementary Movie 6

Supplementary Information Supplementary Movie 7

Supplementary Information Supplementary Movie 8

Supplementary Information Supplementary Movie 9

Supplementary Information Supplementary Movie 10
