# Supplementary material for: Dissection of keratin network formation, turnover and reorganization in living murine embryos
Source: Sci Rep. 2015 Mar 11;5:9007. doi: 10.1038/srep09007 (PMC4355630; doi:10.1038/srep09007)
Supplement: Supplementary Information [file srep09007-s1.pdf]

Dissection of keratin network formation, turnover and reorganization in living murine embryos

Nicole Schwarz[1], Reinhard Windoffer[1], Thomas M. Magin[2], Rudolf E. Leube[1,*]

Supplementary information

Legends to Supplementary Figures

Supplementary Figure 1 | Micrographs of hematoxylin-eosin stained tissue sections of homozygous Krt8-YFP knock-in mice. Liver (a, a'), kidney (b, b'), small intestine (c, c') and trachea (d, d') of 5 week old (a, b, c, d) and 50 week old (a', b', c', d') female mice are shown. No obvious defects are observed. Scale bars, 100 µm.

Supplementary Figure 2 | Full-length blots and gels corresponding to Figure 1b and Figure 2a.

Legends to Supplementary Movies

Supplementary Movie 1 | Time-lapse fluorescence recording (15-minute recording intervals) of an embryoid body derived from ESC clone 72 containing a Krt8-YFPneo allele (corresponding Figure 1 e-e'''). Note that only the outer cell layer corresponding to the primitive endoderm shows filamentous fluorescence in the cytoplasm. Some of these highly dynamic cells move outward upon attachment of the embryoid body to the gelatin-coated glass bottom dish. Scale bar, 50 µm.

Supplementary Movie 2 | Animation of a stack of 11 confocal planes recording the Krt8-YFP fluorescence in a non-fixed intestinal villus tip (see also corresponding Figure 3a-a''). Note the subapically and laterally restricted network distribution. Scale bar, 10 µm.

Supplementary Movie 3 | Animation of a stack of 13 confocal planes recording the Krt8-YFP fluorescence in non-fixed intestinal epithelial cells (see also corresponding Figure 3b-b'). The oblique views show details of the subapical and submembraneous keratin filament network. Scale bar, 10 µm.

Supplementary Movie 4 | Animation of 3D-reconstruction corresponding to Figure 4c depicts the Krt8-YFP fluorescence in the outer trophectoderm of a late blastocyst. Note the punctate accumulation at the plasma membranes in association with the developing cytoplasmic keratin network. The relative fluorescence intensity is color coded. Scale bar, 20 µm.

Supplementary Movie 5 | Time-lapse imaging of Krt8-YFP fluorescence in a developing homozygous knock-in 8-cell embryo (corresponding Figure 5b-b"). Recording intervals, 30 minutes. Note that diffuse cytoplasmic fluorescence starts to appear around compaction (7 h) and that granular structures emerge subsequently at cell-cell borders. Scale bar, 20 µm.

Supplementary Movie 6 | Time-lapse fluorescence microscopy of compacted homozygous Krt8-YFP morula at the transition to the blastocyst stage (corresponding Figure 5d-d"). Recording intervals, 15 minutes. The movie depicts a continuous increase of fluorescent puncta at cell-cell contact regions and highly motile filamentous structures in the cytoplasm. Scale bar, 20 µm.

Supplementary Movie 7 | Time-lapse recording of Krt8-YFP fluorescence in a mid-blastocyst (corresponding Figure 5f-f"; recording intervals, 210 seconds). The highly flexible cytoplasmic particles move throughout the cytoplasm at random. They fuse occasionally but also separate into smaller fragments. In contrast, the dotted cell border-restricted fluorescence is rather stable. Scale bar, 10 µm.

Supplementary Movie 8 | Time-lapse fluorescence microscopy of Krt8-YFP in a late blastocyst (corresponding Figure 5h-h"; recording intervals, 60 seconds). The extended cytoplasmic filament network and the cell border fluorescence both remain stable with only little fluctuation. Scale bar, 10 µm.

Supplementary Movie 9 | Time-lapse fluorescence microscopy of Krt8-YFP in a mid-blastocyst depicting keratin rearrangements during mitosis (corresponding Figure 7c-c"'; recording intervals, 5 minutes). Note the transient increase in diffuse fluorescence at the onset of mitosis and its reduction at the end of mitosis. Scale bar, 10 µm.

Supplementary Movie 10 | Time-lapse recording of Krt8-YFP fluorescence from the compacted morula to the blastocyst stage highlighting keratin reorganization during cell division (corresponding Figure 2d-d'''; recording interval, 15 minutes). The first part of the video is identical to Supplementary Movie 6. Note the transient increase in diffuse fluorescence and the fragmented appearance of the network during pro-metaphase and the rapid formation of an extended filament network during ana-telophase. Mitotic cells are labeled with triangle and asterisk. Scale bar, 10 µm.

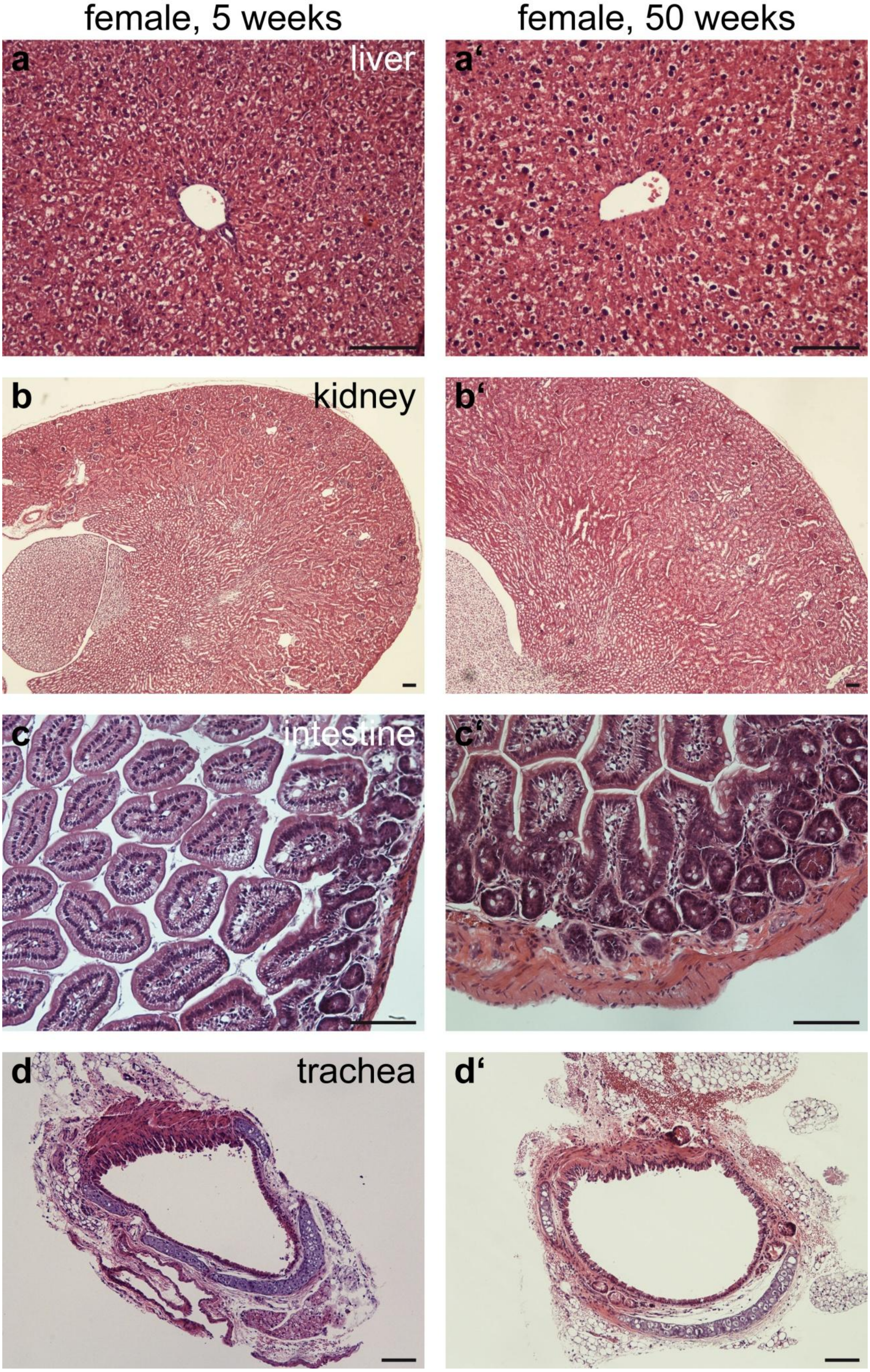

Supplementary Figure 1

Fig. 1b

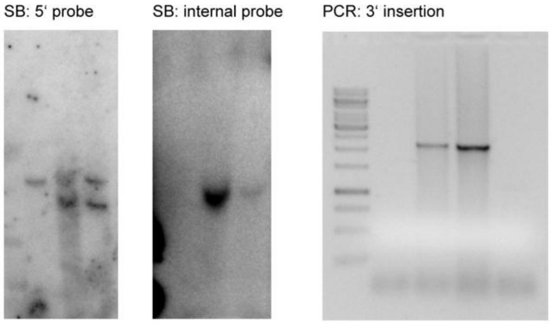

Fig. 2a

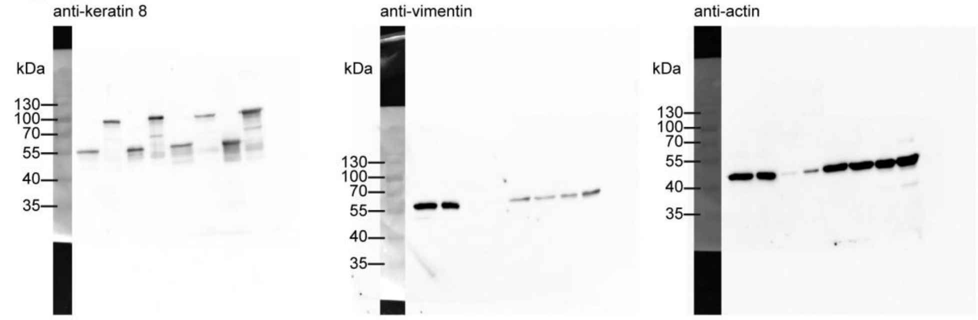

Supplementary Figure 2
